# Temporal stability of optimism and pessimism (LOT-R) over 6 years in the general population

**DOI:** 10.3389/fpsyg.2024.1379651

**Published:** 2024-06-20

**Authors:** Andreas Hinz, Michael Friedrich, Heide Glaesmer, Barbara Brendel, Yuriy Nesterko, Jochen Ernst, Matthias L. Schroeter, Anja Mehnert-Theuerkauf

**Affiliations:** ^1^Department of Medical Psychology and Medical Sociology, University of Leipzig, Leipzig, Germany; ^2^Max Planck Institute for Human Cognitive and Brain Sciences, Leipzig, Germany; ^3^Clinic for Cognitive Neurology, University Hospital Leipzig, Leipzig, Germany

**Keywords:** optimism, pessimism, longitudinal study, temporal stability, reliability

## Abstract

**Objective:**

The aims of this study were to examine changes in habitual optimism over a six-year period and to analyze the relationship between changes in optimism and changes in other quality of life-related variables.

**Method:**

A randomly selected community sample of the German adult general population (*N* = 4,965) was surveyed twice, with a time interval of 6.04 years.

**Results:**

During the course of the 6 years, the mean score of the LOT-R total scale improved (effect size *d* = 0.11). The temporal stability in terms of the test–retest correlation was *r* = 0.61 for the total sample. There were only marginal gender differences in this temporal stability, however, the stability in the oldest age group ≥70 years (*r* = 0.50) was lower than the stability of the other age groups. The cross-sectional correlations showed clear relationships between optimism on the one hand and quality of life, life satisfaction, social support, and low levels of anxiety and physical complaints on the other. The corresponding longitudinal correlations between changes in optimism and changes in the other variables were less pronounced, but in the same direction.

**Conclusion:**

The study confirmed the applicability of the LOT-R in longitudinal studies. In samples with participants of 70 years and above, the limited stability in the optimism assessments needs to be considered in clinical practice and epidemiologic research.

## 1 Introduction

Dispositional optimism is a personality trait that reflects the general tendency to expect that good things will happen in the future (Scheier and Carver, 1992). Like concepts such as self-efficacy, resilience, resilient coping, and sense of coherence, optimism can be understood as a resource variable that buffers aversive life events. Optimism is associated with mental and physical health (Schou-Bredal et al., 2019), quality of life (Liu et al., 2021; Marton et al., 2022), education and physical activity (Craig et al., 2023), positive adjustment to stressful life events and coping (Ramírez-Maestre et al., 2019), life satisfaction (Cerezo et al., 2022), spirituality (Ciria-Suarez et al., 2021), low levels of anxiety and depression (Faye-Schjøll and Schou-Bredal, 2019; Menéndez-Aller et al., 2020), low risk of burnout (Tutte-Vallarino et al., 2022), low perceived risk for COVID-19 (Schou-Bredal et al., 2021), and even lower mortality (Liu et al., 2022a). The relevance of optimism has been shown in several clinical areas such as heart diseases (Huffman et al., 2019), cancer (Liu et al., 2022b; Hinz et al., 2023), gynecology (Morán-Sánchez et al., 2021), obstetrics (Giangiordano et al., 2020), and chronic pain (Esteve et al., 2018). Recently, optimism (and pessimism) have been discussed as anticipatory feelings related to the process of anticipation and prediction of future events (Stefanova et al., 2020).

The most frequently used questionnaire for measuring optimism is the Life Orientation Test-Revised (LOT-R; Scheier et al., 1994). It consists of two subscales, optimism and pessimism. Multiple studies have been performed that tested the two-dimensional structure of the questionnaire (Cano-García et al., 2015; Hinz et al., 2017; Huang et al., 2020). Until now, it is a matter of debate whether it is justified to combine the two subscales of the LOT-R to one total score (Cano-García et al., 2015; Hinz et al., 2022b). While the optimism and the pessimism subscales are only weakly negatively correlated (Glaesmer et al., 2012; Hinz et al., 2022b), the correlations of the total score with other related constructs are generally somewhat higher than the correlations of the subscales (Hinz et al., 2022b), which is an argument for using this total score.

Normative values for the LOT-R are available from several countries (Zenger et al., 2013; Hinz et al., 2017; Schou-Bredal et al., 2017). These normative studies also include analyses of age and gender differences in optimism in general population samples. However, the knowledge about long-term stability in optimism and the temporal stability of this construct is limited (Armbruster et al., 2015; Saboonchi et al., 2016). Changes in mental health or in resource variables such as optimism can be considered from two different perspectives: mean score changes from t1 to t2, and test–retest correlations which indicate the individual stability, i.e., the stability of the rank positions of the participants. It is well-known that the temporal stability of personality traits is somewhat higher than the stability of health-related variables (Anusic and Schimmack, 2016; Struijs et al., 2020). However, a thorough investigation of long-term changes in optimism, including age and gender differences in temporal stability, has not been done so far.

As mentioned above, habitual optimism is correlated with multiple other health-related variables. However, it is also relevant to investigate not only cross-sectional but also longitudinal associations: To what degree do changes in optimism correspond with changes in other variables? There are only few studies that investigated such associations between change scores of mental health variables (Kuehner, 2002; Hajek and König, 2016), and such change score correlations have not been applied to optimism yet.

In 2017, the results of a study with the LOT-R were published, based on a large representative community sample of 9,711 persons (Hinz et al., 2017). Six years after this baseline assessment (t1), a follow-up-study (t2) was performed which also included the LOT-R. Based on the data of this longitudinal study, the aims of this article were (a) to investigate changes in habitual optimism over a 6-year period, (b) to analyze gender and age differences in the stability of the LOT-R scores, and (c) to investigate the relationship between changes in the LOT-R score and changes in other health-related variables.

## 2 Methods

### 2.1 Sample of participants

The LIFE-Adult Study, conducted by the Leipzig Research Center for Civilization Diseases (LIFE), was a large population-based study in the city of Leipzig, Germany and a collaboration between several clinical and epidemiological research teams. Between 2011 and 2014, a total of 10,000 persons, ranging in age from 18 to 80 years, were recruited by applying age- and gender-stratified random selection on data obtained from the local residents’ registry office, with a focus on the age range 40–80 years according to the study protocol. The central objective of this study was to examine causes for the development of civilization diseases. Pregnancy and insufficient command of the German language were the only exclusion criteria. At the study center, the participants underwent several assessment batteries, including collection of sociodemographic variables, medical history, psychological variables, and several medical examinations. Sociodemographic factors were obtained in a structured interview. Details of the study protocol and the assessments have been published elsewhere (Loeffler et al., 2015; Engel et al., 2023). The response rate of this LIFE-Adult study (first wave) was 33%. The LOT-R and several other questionnaires were also included in this study. Results of the LOT-R investigation of the first wave of the LIFE-Adult study have already been published (Hinz et al., 2017).

Between 2017 and 2021, a follow-up study was performed. All available participants of the t1 baseline assessment were invited to complete several questionnaires and to answer questions concerning their health status. Both, the baseline (t1) and the follow-up (t2) study, were approved by the Ethics Committee of the Medical Faculty of the University of Leipzig (approval numbers 263–2009-14122009, 263/09-ff, and 201/17-ek), and written informed consent was obtained from all participants.

### 2.2 Instruments

The Life Orientation Test-Revised (LOT-R; Scheier et al., 1994) was designed to measure habitual optimism. The test comprises 10 items: three items are phrased in an optimistic direction (scale optimism), three in a pessimistic direction (scale pessimism), and the remaining four items are neutral filler items. Answer options for each item range from 0 (strongly disagree) to 4 (strongly agree), which results in scale ranges from 0 to 12 for the two single scales (optimism and pessimism), and from 0 to 24 for the total scale, which is composed of the optimism scale and the inverted pessimism scale.

In addition to the LOT-R, the following instruments were used both at baseline and at follow-up: the Generalized Anxiety Disorder screener (GAD-7; Spitzer et al., 2006; Toussaint et al., 2020), the Patient Health Questionnaire-15 (PHQ-15; Kroenke et al., 2002; Cano-García et al., 2020) for measuring bodily complaints, the Satisfaction with Life Scale (SWLS; Diener et al., 1985; Kusier and Folker, 2021), the ENRICHD Social Support Instrument ESSI (Berkman et al., 2003), and the quality of life questionnaire Short Form Health Survey-8 (SF-8; Ware et al., 2001).

### 2.3 Statistical analysis

Mean score differences between two groups of participants were expressed with effect sizes (Cohen’s *d*), and tested with *t*-tests for independent samples. Cronbach’s α coefficient was used to determine internal consistency. Coefficients of temporal stability were calculated using Pearson correlation coefficients. Psychometric properties of the test were assessed using the common discriminatory power coefficients that indicate the correlation between an item and the part-whole-corrected sum score. In addition, we performed discriminatory power analyses with the change scores. These coefficients indicate to what degree the change in a single item from t1 to t2 corresponds with the change of the scale after removing the item of interest. The effects of gender and age group on the change in optimism were tested with a two-way analysis of variance (ANOVA). All statistics were performed with SPSS version 27.

## 3 Results

### 3.1 Characteristics of the sample

The baseline assessment comprised 10,000 persons, of which 9,711 completed the LOT-R at t1. All available participants were invited to take part in the follow-up study, and 5,668 of them agreed to do so (response rate 57%). The mean time interval between the t1 and the t2 examination was 6.04 years (SD = 0.42 years). Reasons for non-participation at the t2 assessment were: non-response without known reasons (55.4%), refusal (29.0%), death (13.2%), loss to follow-up (1.3%), and inability to participate (1.2%). At both t1 and t2, missing values were handled as follows: If only one item was missing in one of the subscales (Optimism or Pessimism), this was replaced by the rounded mean of the other two items. This resulted in a sample size of 9,711 people at t1. 5,063 people took part in the follow-up examination (t2) and answered at least one item of the LOT-R. Finally, there were 4,965 people who had valid values in the LOT-R in both scales and at both time points according to this criterion. Table 1 presents sociodemographic characteristics of this sample at t1.

**Table 1:** Sociodemographic characteristics of the sample at t1.

	Males (*n* = 2,341)		Females (*n* = 2,624)		Total sample (*n* = 4,965)	
	*n*	%	*n*	%	*n*	%
Age						
Mean (SD)	57.7 (12.5)		56.5 (12.2)		57.1 (12.3)	
Age group						
≤ 39 years	137	5.9	133	5.1	270	5.4
40–49 years	551	23.5	713	27.2	1,264	25.5
50–59 years	502	21.4	617	23.5	1,119	22.5
60–69 years	658	28.1	689	26.3	1,347	27.1
≥ 70 years	493	21.1	472	18.0	965	19.4
Marital Status^a^						
Married, living together	1,601	68.4	1,553	59.2	3,154	63.6
Married, living separately	40	1.7	60	2.3	100	2.0
Never married	409	17.5	420	16.0	829	16.7
Divorced	236	10.1	373	14.2	609	12.3
Widowed	55	2.3	216	8.2	271	5.5
Education^a^						
< 10 years	143	6.2	183	7.0	326	6.6
10–11 years	1,185	51.1	1,482	57.1	2,667	54.3
≥ 12 years	991	42.7	932	35.9	1923	39.1
Occupational status^a^						
Working full time	1,189	51.1	1,056	40.6	2,245	45.6
Working part-time	75	3.2	389	15.0	464	9.4
Unemployed	108	4.6	113	4.3	221	4.5
Retired	918	39.5	972	37.4	1,890	38.4
Other	35	1.5	72	2.8	107	2.2
Socio-economic status^a^						
Low	342	14.6	409	15.6	751	15.1
Medium	1,363	58.3	1,666	63.6	3,029	61.1
High	634	27.1	544	20.8	1,178	23.8

^a^Missing data not reported.

### 3.2 Item characteristics and psychometric properties

Item and scale characteristics of the LOT-R are presented in Table 2. The LOT-R mean total score of the 4,965 participants who completed both the t1 and the t2 examinations was M = 16.65 at t1. Those participants of the t1 examination who did not take part in the t2 examination (dropouts) showed the following LOT-R mean scores: 8.54 ± 2.44 (optimism), 4.73 ± 2.43 (pessimism), and 15.82 ± 3.85 (total score) at t1. This means that the participants who also completed the t2 examination (*n* = 4,965), had a significantly higher LOT-R total score (M = 16.65) than the dropouts, with an effect size of *d* = 0.22 (*p* < 0.001). The dropouts were on average slightly younger then the complete participants, 56.3 ± 12.5 years versus 57.1 ± 12.3 years (*p* = 0.002), and the proportion of women in the group of dropouts was slightly and non-significantly lower (51.8%) than in the group of complete respondents (52.8%; *p* = 0.304).

**Table 2:** Characteristics of the LOT-R items (range 0–4), subscales (range: 0–12), and sum score (range 0–24).

Item / Scale	t1		t2		Difference t2-t1		*d* (t2-t1)	discr. power t1	discr. power t2	discr. power (Δitem, Δscale)	*r_tt_*
	M	(SD)	M	(SD)	M	(SD)					
Optimism Item1	2.79	(1.02)	2.83	(0.99)	0.04	(1.13)	0.04	0.52	0.57	0.32	0.37
Optimism Item2	3.08	(0.96)	3.02	(0.99)	−0.06	(1.01)	−0.06	0.55	0.58	0.34	0.46
Optimism Item3	3.08	(1.04)	3.16	(1.00)	0.08	(1.20)	0.08	0.43	0.51	0.27	0.31
Pessimism Item1	1.55	(1.00)	1.39	(0.99)	−0.16	(1.13)	−0.16	0.37	0.44	0.20	0.36
Pessimism Item2	1.36	(0.93)	1.25	(0.95)	−0.11	(1.08)	−0.12	0.49	0.53	0.29	0.34
Pessimism Item3	1.40	(1.14)	1.29	(1.16)	−0.11	(1.33)	−0.10	0.42	0.46	0.27	0.33
Optimism	8.96	(2.37)	9.01	(2.40)	0.05	(2.37)	0.02	α = 0.68	α = 0.73	α = 0.49	0.51
Pessimism	4.31	(2.32)	3.92	(2.40)	−0.39	(2.42)	−0.16	α = 0.61	α = 0.66	α = 0.41	0.48
LOT-R total	16.65	(3.70)	17.09	(3.97)	0.44	(3.40)	0.11	α = 0.66	α = 0.73	α = 0.37	0.61

d(t1, t2): Effect size of the difference t2 score minus t1 score; discr. Power: discriminative power; discr. Power (Δitem, Δscale): discriminative power for the difference scores (t2 minus t1), all discrimination power coefficients refer to the subscales (optimism or pessimism); r_tt_: test–retest correlation; α: Cronbach’s α.

The LOT-R mean total score of the complete respondents increased from 16.65 at t1 to 17.09 at t2 with an effect size of *d* = 0.11. While the results for the item mean score changes from t1 to t2 of the optimism items were mixed (differences between −0.06 and 0.08), all pessimism items showed a decrease, resulting in a lower pessimism mean score at t2. All α coefficients of the cross-sectional analyses were between 0.61 and 0.73.

While the discrimination power coefficients for the cross-sectional analyses were between 0.37 and 0.58, the corresponding longitudinal coefficients were lower but nevertheless positive (range: 0.20 to 0.34), and the α coefficients of the change scores were also lower than those of the cross-sectional analyses (Table 2).

The right part of Table 2 presents the test–retest correlation coefficients for the items and scales. These temporal stability coefficients of the items were in the range from 0.31 to 0.46. Aggregating across items and subscales increased the test–retest correlations; the highest stability coefficient was obtained for the LOT-R total score (*r* = 0.61).

### 3.3 Gender and age differences in optimism and pessimism

Table 3 shows t1 and t2 mean scores for the LOT-R scales, broken down by gender and by age groups. The t1 means (± SD) of the total sample were 8.96 ± 2.37 (optimism), 4.31 ± 2.32 (pessimism), and 16.65 ± 3.70 (total score).

**Table 3:** Mean scores, effect sizes, and test–retest correlations for the LOT-R scales, broken down by gender and age groups.

	t1		t2		Diff. (t2-t1)		*d*	*p*	*r*_tt_
	M	(SD)	M	(SD)	M	(SD)			
Optimism									
Gender									
Males	8.85	(2.35)	9.07	(2.36)	0.22	(2.31)	0.09	<0.001	0.52
Females	9.05	(2.39)	8.96	(2.43)	−0.09	(2.42)	−0.04	0.059	0.50
Age group									
18–39 years	9.04	(2.30)	9.10	(2.44)	0.06	(2.23)	0.03	0.663	0.56
40–49 years	9.08	(2.28)	9.12	(2.36)	0.05	(2.09)	0.02	0.436	0.59
50–59 years	8.94	(2.35)	9.04	(2.46)	0.09	(2.31)	0.04	0.171	0.54
60–69 years	8.87	(2.51)	9.02	(2.41)	0.15	(2.51)	0.06	0.026	0.48
≥ 70 years	8.92	(2.35)	8.81	(2.33)	−0.12	(2.60)	−0.05	0.126	0.38
Total sample	8.96	(2.37)	9.01	(2.40)	0.05	(2.37)	0.02	0.103	0.51
Pessimism									
Gender									
Males	4.38	(2.27)	3.93	(2.34)	−0.45	(2.42)	−0.20	<0.001	0.45
Females	4.25	(2.36)	3.92	(2.46)	−0.33	(2.42)	−0.14	<0.001	0.50
Age group									
18–39 years	3.71	(2.12)	3.41	(2.26)	−0.30	(2.17)	−0.14	0.024	0.51
40–49 years	4.04	(2.30)	3.57	(2.43)	−0.47	(2.40)	−0.20	<0.001	0.49
50–59 years	4.23	(2.40)	3.81	(2.43)	−0.41	(2.38)	−0.17	<0.001	0.52
60–69 years	4.51	(2.25)	4.00	(2.36)	−0.51	(2.36)	−0.22	<0.001	0.48
≥ 70 years	4.67	(2.33)	4.57	(2.30)	−0.10	(2.63)	−0.04	0.260	0.35
Total sample	4.31	(2.32)	3.92	(2.40)	−0.39	(2.42)	−0.17	<0.001	0.48
LOT-R total									
Gender									
Males	16.48	(3.65)	17.14	(3.84)	0.66	(3.35)	0.18	<0.001	0.60
Females	16.80	(3.74)	17.04	(4.08)	0.24	(3.43)	0.06	<0.001	0.62
Age group									
18–39 years	17.33	(3.72)	17.69	(4.06)	0.36	(3.54)	0.09	0.097	0.59
40–49 years	17.04	(3.83)	17.56	(4.17)	0.51	(3.38)	0.13	<0.001	0.64
50–59 years	16.72	(3.90)	17.23	(4.12)	0.51	(3.40)	0.13	<0.001	0.64
60–69 years	16.36	(3.63)	17.03	(3.83)	0.66	(3.36)	0.18	<0.001	0.60
≥ 70 years	16.25	(3.31)	16.23	(3.51)	−0.02	(3.40)	−0.01	0.842	0.50
Total sample	16.65	(3.70)	17.09	(3.97)	0.44	(3.40)	0.11	<0.001	0.61

d, effect size; *p*, significance level.

Females showed slightly higher LOT-R mean total scores than males did at t1, while at t2 the relationship was reversed. Males gained in optimism (LOT-R total score) from t1 to t2 with an effect size of *d* = 0.18, the corresponding increase of the females was lower (*d* = 0.06).

Both at t1 and at t2 there was an age trend with lowest LOT-R mean total scores for the older age groups. All age groups except those of 70 years and above gained in optimism during the 6-year period as reflected in positive effect sizes in Table 3; the effect size of the difference between the LOT-R total scores at t2 and t1 was *d* = 0.11. Gender and age group differences in the LOT-R total change scores yielded the following ANOVA results: gender (*F* = 14.04, *p* < 0.001), age group (*F* = 6.64, *p* < 0.001), and gender * age group (*F* = 1.61, *p* = 0.169).

The temporal stability (Table 3, right column) was similar for both genders, with total score stability coefficients of *r* = 0.60 and *r* = 0.62 for males and females, respectively. Regarding age, the stability coefficients were similar for all age groups (*r* between 0.59 and 0.64) except the oldest group (≥ 70 years) that showed a lower degree of temporal stability (*r* = 0.50). The age range with the highest temporal stability was that from 40 to 59 years of age.

### 3.4 Correlations with other quality-of-life-related variables

Table 4 presents the correlations between the LOT-R scales and several other scales. For each of the two subscales and also for the total scale, the table shows correlations within t1, e.g., *r* (LOT-Optimism, SWLS) = 0.44, correlations within t2, e.g., *r* (LOT-Optimism, SWLS) = 0.49, and correlations between the change scores (difference between t2 score and t1 score) of LOT-R and the change score of the respective other scale, e.g., *r*(Δ LOT-Optimism, Δ SWLS) = 0.23. All coefficients in Table 4, even those of 0.07, are statistically significant with *p* < 0.001 due to the large sample size. Both optimism and pessimism were most strongly correlated with satisfaction with life, the LOT-R total score reached correlations with the satisfaction with life scores of 0.50 (t1) and 0.54 (t2). In most cases, the t2 correlations were slightly higher than the t1 correlations. All correlations of the LOT-R total score were stronger than the single correlations of the subscales optimism and pessimism.

**Table 4:** Correlations between the LOT-R scales and other scales.

	Optimism			Pessimism			Total		
	r (LOT-Opt, scale) at t1	r (LOT-Opt, scale) at t2	r (Δ LOT-Opt, Δ scale)	r (LOT-Pess, scale) at t1	r (LOT-Pess, scale) at t2	r (Δ LOT-Pess, Δ scale)	r (LOT-Total, scale) at t1	r (LOT-Total, scale) at t2	r (Δ LOT-Total, Δ scale)
SWLS: Life satisfaction	0.44	0.49	0.23	−0.34	−0.40	−0.21	0.50	0.54	0.31
ENRICHD: Social Support	0.27	0.33	0.13	−0.20	−0.29	−0.09	0.30	0.37	0.16
GAD-7: Anxiety	−0.30	−0.40	−0.18	0.28	0.37	0.16	−0.37	−0.46	−0.24
PHQ-15: Complaints	−0.23	−0.36	−0.11	0.22	0.33	0.13	−0.29	−0.41	−0.17
SF-8: Physical functioning	0.19	0.25	0.08	−0.20	−0.26	−0.09	0.25	0.31	0.12
SF-8: Role-physical	0.21	0.25	0.10	−0.19	−0.25	−0.08	0.25	0.31	0.13
SF-8: Bodily pain	0.14	0.20	0.04	−0.12	−0.21	−0.07	0.17	0.25	0.07
SF-8: General health	0.28	0.31	0.10	−0.23	−0.29	−0.07	0.32	0.36	0.12
SF-8: Vitality	0.31	0.34	0.13	−0.22	−0.29	−0.09	0.33	0.38	0.15
SF-8: Social functioning	0.27	0.30	0.13	−0.22	−0.29	−0.12	0.31	0.36	0.17
SF-8: Role-emotional	0.26	0.29	0.12	−0.22	−0.26	−0.13	0.30	0.33	0.17
SF-8: Mental health	0.31	0.33	0.17	−0.22	−0.28	−0.12	0.33	0.37	0.20
SF-8: PCS	0.18	0.23	0.07	−0.18	−0.24	−0.08	0.23	0.29	0.10
SF-8: MCS	0.33	0.35	0.17	−0.22	−0.29	−0.13	0.35	0.39	0.21

Opt, optimism; Pess, pessimism; PCS, Physical Component Summary; MCS, Mental Component Summary; Δ, difference between the t2 and the t1 score.

Regarding the correlations of the change scores, all correlations were weaker than those of the raw scores, but they were in the same direction. Participants who gained in satisfaction with life from t1 to t2 also gained in optimism (*r* = 0.21) and lost in pessimism (*r* = −0.21); the association to the LOT-R total score was stronger (*r* = 0.31). The sequence of the correlations (with highest scores for satisfaction with life and the MCS component of the SF-8, and lowest scores for bodily pain and the PCS score of the SF-8) was very similar for the raw scores and the change scores.

The correlations between the optimism and the pessimism subscales were *r* = −0.24 (at t1), *r* = −0.36 (at t2), and *r* = −0.01 (change scores).

## 4 Discussion

The main objective of this study was to investigate the course and the temporal stability of the LOT-R scores over a 6-year period. The LOT-R mean score increased from 16.65 to 17.09 (*d* = 0.11). This improvement is in line with a study that detected an improvement of mental health (as measured with the SF-8) during a 5-year period (Hopman et al., 2006), but it contradicts other studies which found decreases in mental health (Hemingway et al., 1997). In this context, it is interesting to note that cross-sectional studies on the distribution of optimism showed a decrease in optimism with increasing age (Hinz et al., 2017), which indicates that age dependencies, when derived from cross-sectional studies, can result in other conclusions than those of longitudinal studies which aggregate over individual changes. Further research is needed to clarify the conditions under which long-term increases or decreases of mental health variables occur.

There was a gender effect in the changes of the LOT-R scores: The improvement of the total sample (diff = 0.44) was mainly due to an improvement of the males (diff = 0.66), while the females showed only a slight increase (diff = 0.24). Regarding age, there was a clear and non-linear effect of age group on the LOT-R change score. While the oldest age group (70 years and above) remained relatively stable in the LOT-R mean score level, all other age groups showed an increase, and the strongest increase was found for the age group 60–69 years, which may be due to the beginning of retirement (Prakash et al., 2022; Henning et al., 2023). A decrease in mental health in the oldest age group (75 years and above) was also found in a general population study with a five-year interval (Hopman et al., 2006). Since the increases or decreases in optimism do not follow a linear age trend, statistics based on linear age dependencies are insufficient tools for describing such changes. Particular attention should be given to people in the age group of 70 years and above.

The temporal stability in terms of test–retest correlations of the LOT-R total score was *r* = 0.61 for the total sample. There are findings on the temporal stability of the LOT-R in several samples of patients, with coefficients between 0.60 to 0.69, e.g., (Hinz et al., 2022b), however, it is problematic to compare these coefficients with those of our study since the time interval was shorter in the patients’ samples (in most cases 3 months). Furthermore, in groups of patients, the individual fluctuations in optimism and mental health will be higher than in the general population due to the different courses of the diseases. A study with university students found a test–retest correlation of *r* = 0.74, based on a relatively short time interval of only 5 weeks (Trottier et al., 2008), which is understandably higher than the coefficient obtained in our study. Comparisons of such stability coefficients should always take into account the characteristics of the sample and the time interval between the measurements.

While there were only small differences in temporal stability between males and females in general, there were remarkable age differences. The highest temporal stability was found for the age range 40–59 years, and the most pronounced fluctuations were observed for the oldest group (70 years and above). This means that beginning at the age of about 70 years, the prognoses of the individual well-being and optimism become superimposed by other effects.

For all gender and age groups, the stability coefficients of the LOT-R total score were higher than those of the subscales optimism and pessimism. This also confirms that the combination of optimism and lack of pessimism into a sum score is useful, even if CFAs indicate the preference for a two-dimensional model.

The LOT-R scores were statistically associated with multiple other quality-of-life-related variables. As was to be expected, the association between the LOT-R score and satisfaction with life was relatively strong (*r* = 0.50 at t1), which is comparable with the coefficient (*r* = 0.45) obtained in another general population study with a similar instrument for measuring life satisfaction (Glaesmer et al., 2012). The associations of the LOT-R total scores with the Mental Component Score of the SF-8 were higher than those with the Physical Component Score both at t1 and at t2 as well as regarding the change score, which underlines the strong relationship between optimism and the mental component of QoL even in the longitudinal perspective. This result is also consistent with the finding that LOT-R optimism captures a positive current state of mind nearly just as strongly as it captures the expectation of future positive events (Hinz et al., 2022a).

A secondary finding was that the correlations obtained in the t2 examination were generally somewhat higher than those of the t1 assessment (Table 4). Evidently, the t2 assessments were somewhat more consistent than those of t1, which is also reflected in slightly higher Cronbach α coefficients at t2 in comparison to the t1 α coefficient. This higher consistency at t2 may be due to the fact that the participants completed the LOT-R at the study center during their t1 examination, while at t2 they completed it at home, which might have contributed to a more consistent way of answering the questions.

In the context of the longitudinal analysis, it is interesting to relate changes in optimism to changes in the QoL and mental health variables. All correlations of the change scores were in the same direction as the cross-sectional correlations; increases in the LOT-R score were associated with increases in life satisfaction, social support, and all components of QoL as well as with decreases in anxiety and in bodily complaints. All these correlation coefficients were lower than the cross-sectional coefficients, a result which has also been found in other studies using such correlations of change scores (Kuehner, 2002; Hajek and König, 2016). Causal interpretations of the correlations of the change scores are not possible: an increase in optimism may be a consequence of a decrease in bodily complaints, and vice versa. A special feature of change score correlations compared to cross-sectional correlations is that they are not affected by response sets. While the positive cross-sectional correlations between two health variables can be, at least in part, due to certain response sets or acquiescence tendencies (Hibbing et al., 2019), these possible response tendencies cancel each other out when individual change scores are calculated as the differences between individual scores.

Some limitations of this study should be mentioned. The sample of the study participants was not perfectly representative of the general population. First, the participants of the baseline assessment were slightly healthier than those who refused participation at t1 (Enzenbach et al., 2019). Second, the comparison between those who dropped out and those who completed both the t1 and the t2 assessment showed an additional bias toward healthy participants. This is a general problem in longitudinal studies and limits the generalizability of the mean score levels, however, the changes over time which are the main topic of this study are less affected by such bias effects. A further general problem of the LOT-R is that both subscales are only weakly negatively correlated, and that the calculation of a sum score may therefore be criticized. Nevertheless, we believe that the superiority of the sum score over the subscales in terms of higher Cronbach’s α coefficients, higher stability coefficients, and higher correlations with the other QoL scales justifies the use of this sum score.

The LOT was designed as an instrument to measure habitual optimism in terms of a stable personality trait. This raises the question of whether such an instrument is suitable at all for recording changes. Recently, a State Optimism Questionnaire (SOM) was presented (Hoeppner et al., 2022), which was tailored to detect individual changes in optimism. However, we believe that the LOT-R is nevertheless the more suitable method, because this questionnaire enables both the assessment of states and changes (by means of differences) in optimism and thus also the comparison of cross-sectional and longitudinal correlations. In addition, unlike the SOM, there are LOT-R norm values and extensive psychometric tests.

Taken together, the results of this study show the degree to which mean score changes and individual score changes of optimism and pessimism occur in the general population. These data can be used to better interpret health changes in samples of patients. People or patient groups older than 70 years deserve special attention because of the limited temporal stability of their optimism scores.

## Data availability statement

The datasets presented in this article are not readily available because data cannot be shared publicly because of data protection reasons. Data availability is only possible via a project agreement. Data are available from the LIFE-Adult Study Institutional Data Access / Ethics Committee (contact via https://ldp.life.uni-leipzig.de/) for researchers who meet the criteria for access to confidential data. Requests to access the datasets should be directed to https://ldp.life.uni-leipzig.de.

## Ethics statement

The studies involving humans were approved by Ethical Review Board of the Leipzig University. The studies were conducted in accordance with the local legislation and institutional requirements. The participants provided their written informed consent to participate in this study.

## Author contributions

AH: Validation, Writing – original draft, Writing – review & editing. MF: Formal analysis, Writing – review & editing. HG: Investigation, Writing – review & editing. BB: Visualization, Writing – original draft. YN: Data curation, Methodology, Writing – review & editing. MS: Investigation, Writing – review & editing. JE: Conceptualization, Data curation, Writing – review & editing. AM-T: Resources, Writing – original draft.
